# Eight-and-a-Half Syndrome Secondary to Acute Brainstem Infarction

**DOI:** 10.7759/cureus.65138

**Published:** 2024-07-22

**Authors:** Hamizah Muhammad, Wei Sheng Chan, Juanarita Jaafar, Wan-Hazabbah Wan Hitam

**Affiliations:** 1 Department of Ophthalmology and Visual Science, School of Medical Sciences, Universiti Sains Malaysia, Kubang Kerian, MYS; 2 Department of Ophthalmology and Visual Science, Hospital Universiti Sains Malaysia, Kubang Kerian, MYS; 3 Department of Ophthalmology, Hospital Sultanah Bahiyah, Alor Setar, MYS

**Keywords:** brain magnetic resonance, unilateral facial nerve palsy, brainstem stroke, internuclear ophthalmoplegia, eight-and-a-half syndrome

## Abstract

Eight-and-a-half syndrome is a rare neuro-ophthalmologic condition, which is characterized by ipsilateral horizontal gaze palsy, internuclear ophthalmoplegia (INO), and ipsilateral lower motor neuron facial palsy. We report a case of eight-and-a-half syndrome secondary to acute brainstem infarction.

A 55-year-old gentleman with underlying diabetes mellitus and hypertension presented with a sudden onset of double vision in the right lateral gaze for one day. On examination, there was a limitation in the left eye horizontal eye movement with limited right eye adduction. Further neurological examination revealed left lower motor neuron facial nerve palsy. Magnetic resonance imaging (MRI) of the brain showed an acute infarct involving the left side of the thalamus extending to the left side of the midbrain, pons, and medulla. He was diagnosed with eight-and-a-half syndrome secondary to acute brainstem infarction. The patient was referred to the neuromedical team, where he was treated with anti-platelet medications. He showed gradual improvement on follow-up and had complete resolution of ophthalmoplegia after three months. There was only minimal residual facial nerve weakness.

Eight-and-a-half syndrome has a localizing value to the dorsal tegmentum of the pons. It requires thorough neurological examination and imaging studies for accurate diagnosis and management. This case highlights the potential for a significant recovery in patients with eight-and-a-half syndrome when timely and appropriate treatment is administered.

## Introduction

Eight-and-a-half syndrome is a rare neuro-ophthalmological condition characterized by a unique combination of clinical signs resulting from a lesion in the pons of the brainstem. It is characterized by ipsilateral horizontal gaze palsy and contralateral internuclear ophthalmoplegia (INO) with ipsilateral lower motor neuron facial palsy.

First described by Eggenberger in 1998, this distinctive clinical presentation arises from lesions affecting specific neuroanatomical structures within the pontine tegmentum [1,2]. Acute brainstem infarction, particularly involving the pontine region, can give rise to a variety of complex neurological syndromes due to the dense aggregation of critical neural pathways and nuclei in this area [3]. The paramedian pontine reticular formation (PPRF), abducens nucleus, medial longitudinal fasciculus (MLF), and facial nerve nucleus or its fascicles can all be implicated in such vascular events, leading to the distinctive clinical manifestations of the eight-and-a-half syndrome. We report a case of left-sided eight-and-a-half syndrome secondary to acute brainstem infarction.

## Case presentation

A 55-year-old gentleman with underlying diabetes mellitus and hypertension presented with a sudden onset of double vision in the right lateral gaze for one day. It was associated with dizziness and vomiting. Otherwise, there was no limb weakness and no abnormal gait. 

**Figure 1:**
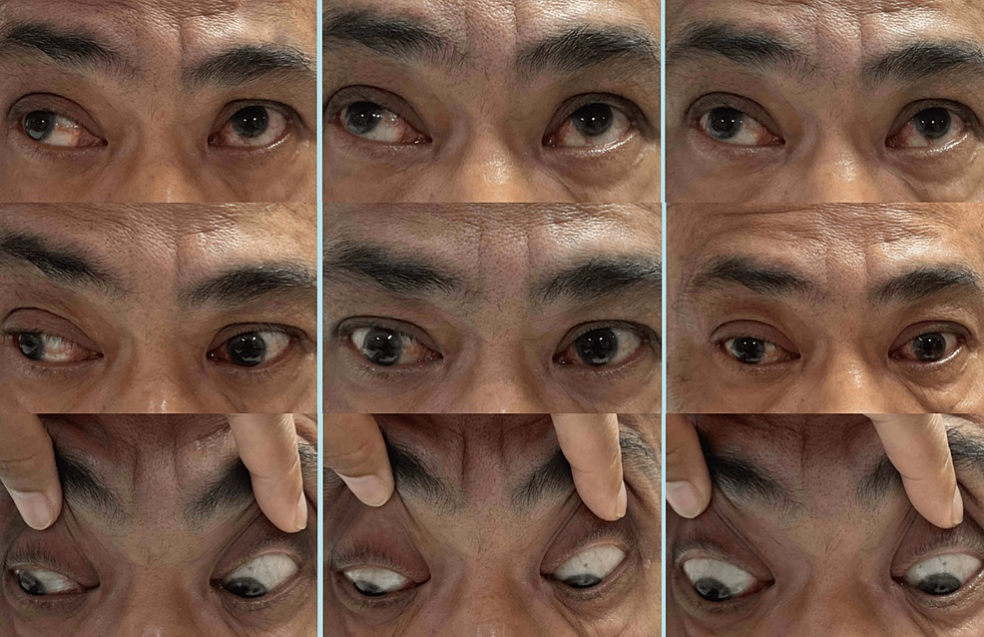
Left eye horizontal gaze palsy with right eye limited adduction

On examination, the visual acuity of both eyes was 6/36. The relative afferent pupillary defect was negative. On primary gaze, the light reflex of the right eye was displaced nasally amounting to 30 degrees of exotropia. There was limited abduction and adduction in his left eye with limited adduction in his right eye (Figure 1). His convergence to near fixation was intact. His anterior segment examination revealed a nuclear sclerosis cataract; otherwise other findings were unremarkable. His posterior segment examination was normal.

**Figure 2:**
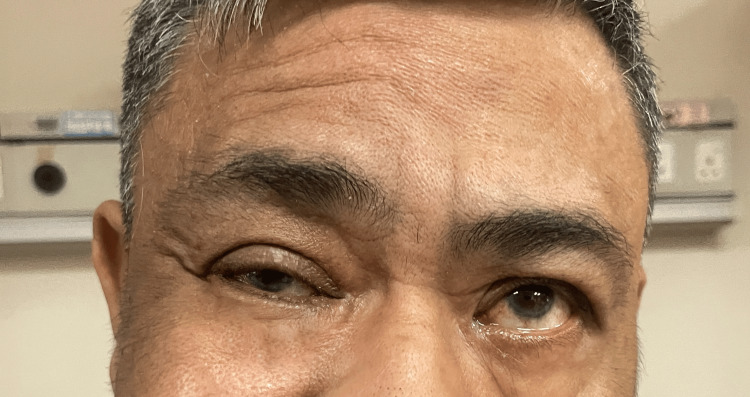
Loss of left forehead wrinkle

Further neurological examination revealed left lower motor neuron facial palsy demonstrated by loss of left forehead wrinkle (Figure 2) and left nasolabial fold with drooping of the left side of the mouth. 

**Figure 3:**
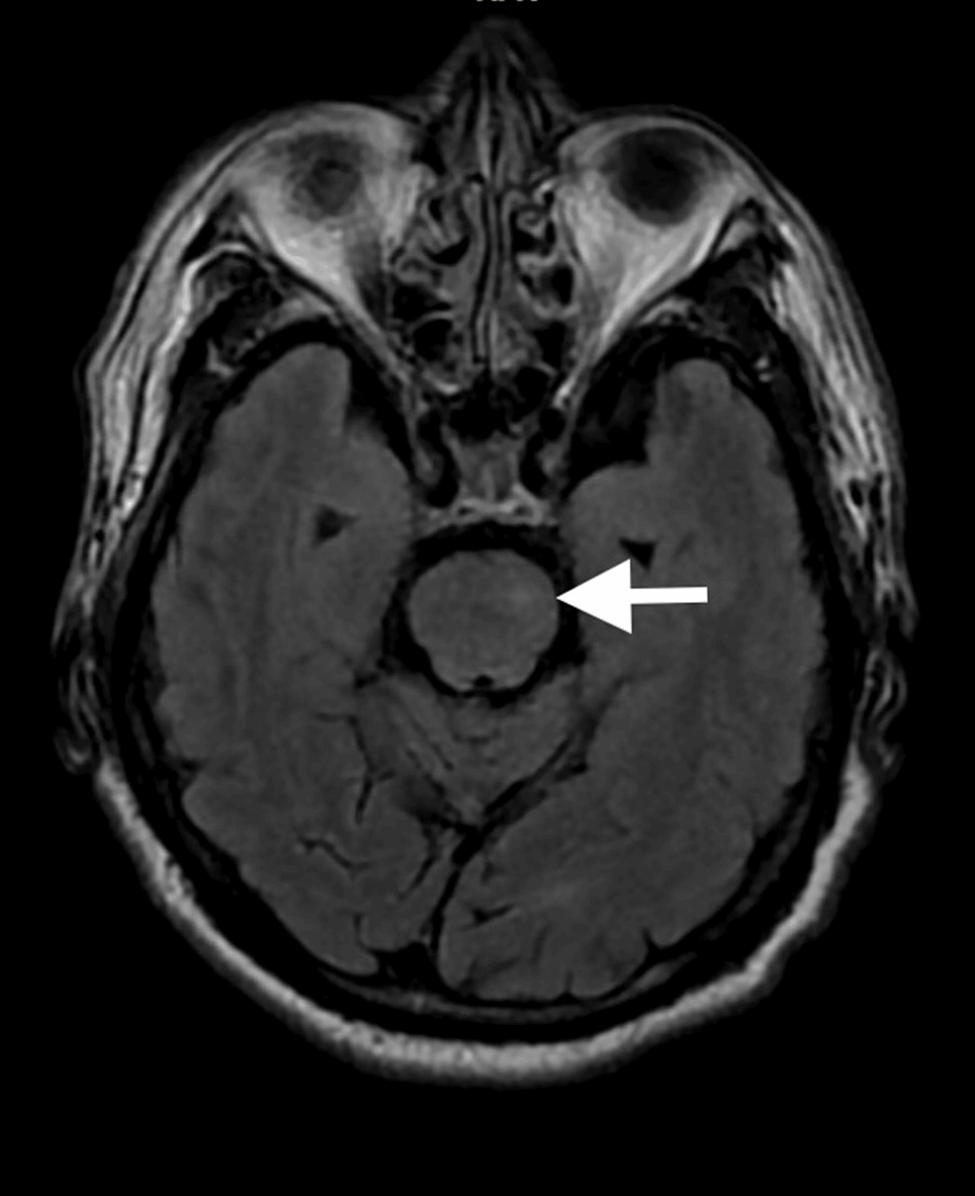
MRI brain showed FLAIR hyperintense lesion (arrow) at the level of left pons MRI, magnetic resonance imaging; FLAIR, fluid-attenuated inversion recovery

**Figure 4:**
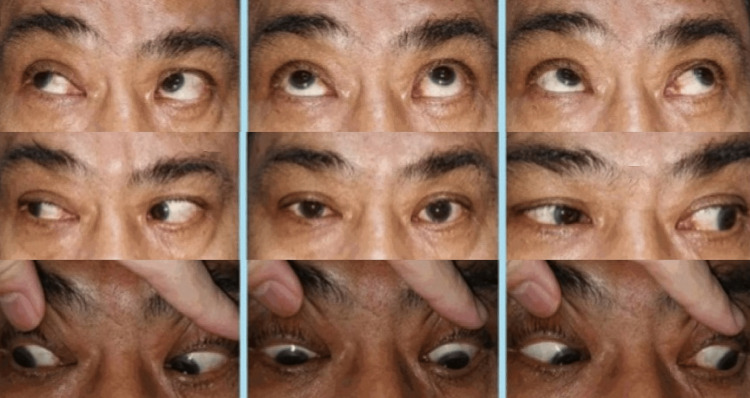
Resolved ophthalmoplegia on subsequent follow-up

His brain CT scan showed no abnormality. Magnetic Resonance Imaging (MRI) of the brain showed an acute infarct involving the left side of the thalamus extending to the left side of the midbrain, pons, and medulla (Figure 3). He was diagnosed with eight-and-a-half syndrome secondary to acute brainstem infarction and was referred to the neuromedical team, where he was treated with anti-platelet medications. He showed gradual improvement on follow-up and had complete resolution of ophthalmoplegia after three months (Figure 4). There was only minimal residual facial nerve weakness. 

## Discussion

The control of horizontal gaze is located in the brainstem and involves a few structures and pathways. The paramedian PPRF plays a pivotal role in generating conjugate horizontal eye movement by integrating inputs from the contralateral frontal eye field (FEF). The impulse is subsequently transmitted from the ipsilateral abducens nucleus to the contralateral oculomotor nucleus via MLF, resulting in a conjugate horizontal gaze [4]. A lesion affecting the medial MLF will result in INO, characterized by impaired eye adduction on the affected side and abduction nystagmus of the contralateral eye [5]. 

Damage to both PPRF and MLF on one side results in a neurological condition known as one-and-a-half syndrome. This condition is a combination of horizontal gaze palsy in one eye and INO in the other eye. Rarely, if the lesion extends to the ipsilateral facial nerve nucleus or its surrounding fascicles, it can lead to ipsilateral lower motor neuron facial nerve palsy. When one-and-a-half syndrome coexists with lower motor neuron facial nerve palsy, it is referred to as eight-and-a-half syndrome [6].

Eight-and-a-half syndrome has a localizing value to the dorsal tegmentum of the pons, where horizontal gaze center structures, facial nucleus, and fascicles are located [4]. The incidence of eight-and-a-half syndrome is relatively rare, with cases most often reported in medical literature rather than large epidemiological studies. Due to its rarity, precise incidence rates are not well-established, but it is generally considered an uncommon neurological condition. The most common cause of this syndrome is cerebrovascular disease, including ischemic stroke and intracranial hemorrhage, followed by demyelinating disease [7]. Others include tuberculoma and intracranial capillary telangiectasia [8,9]. 

Brainstem infarctions can present with a wide range of clinical features depending on the specific location and extent of the infarction. Features of brainstem infarctions include gait ataxia, dysarthria, hemiparesis, and INO depending on the site of the lesion [3]. Our case specifically involved the rare combination of symptoms defining eight-and-a-half syndrome without any other systemic symptoms. 

The initial evaluation of patients with suspected acute stroke often relies on non-contrast CT scans due to their widespread availability and ability to quickly rule out hemorrhage. However, it has relatively low sensitivity for early detection of acute ischemic stroke [10]. MRI, conversely, is highly sensitive, specific, and accurate in identifying acute ischemic changes [10]. In our patient, it is noteworthy that while the initial CT brain was inconclusive, a subsequent MRI revealed an acute brainstem infarction. This underscores the importance of thorough imaging, such as MRI, in cases where clinical suspicion remains high despite negative initial findings on CT scan; thus, early and appropriate treatment can be initiated. 

The prognosis of eight-and-a-half syndrome largely depends on the timely and appropriate treatment of the underlying cause. Recovery from a brainstem stroke can be a long and challenging process. A follow-up review of our patient revealed complete recovery of his ophthalmic symptoms with some minimal residual facial nerve weakness, consistent with findings from previous case reports [11,12]. 

## Conclusions

Eight-and-a-half syndrome is a rare neuro-ophthalmologic condition, which has a localizing value to the dorsal tegmentum of the pons. Given its rarity, eight-and-a-half syndrome provides valuable insights into the anatomy and vascular supply of the brainstem and highlights the need for prompt and accurate diagnosis to manage potential complications and optimize patient outcomes. It requires thorough neurological examination and imaging studies for accurate diagnosis and management. This case highlights the potential for a significant recovery in patients with eight-and-a-half syndrome when timely and appropriate treatment is administered.
